# Concentrated growth factor promotes proliferation, osteogenic differentiation, and angiogenic potential of rabbit periosteum-derived cells in vitro

**DOI:** 10.1186/s13018-019-1164-3

**Published:** 2019-05-22

**Authors:** Lili Zhang, Hongjun Ai

**Affiliations:** 1 Department of Prosthodontics, School of Stomatology, China Medical University, No. 117, Nanjing North Street, Heping District, Shenyang, Liaoning 110002 People’s Republic of China; 2 Department of Stomatology, First Affiliated Hospital of Jinzhou Medical University, Jinzhou, 121000 Liaoning China

**Keywords:** Concentrated growth factor, Periosteum-derived cells, Bone tissue engineering

## Abstract

**Objective:**

The aim of this research is to investigate the effects of concentrated growth factor (CGF) on the proliferation, osteogenic differentiation, and angiogenic potential of rabbit periosteum-derived cells (PDCs) in vitro.

**Methods:**

PDCs were isolated from the femoral and tibial periosteum of rabbits and cultured with or without CGF membranes or CGF conditioned media. Scanning electron microscopy (SEM) was used for the structural characterization. Cell Counting Kit-8 assay was used to measure cell proliferation. Alkaline phosphatase (ALP) activity of PDCs was also measured. Immunohistochemistry was used to detect the expression of CD34. Enzyme-linked immunosorbent assay (ELISA), quantitative real-time PCR (qPCR), and Western blot were used to evaluate the secretion and expression levels of osteogenic differentiation markers (bone morphogenetic protein-2, type I collagen, osteocalcin) and angiogenesis markers (vascular endothelial growth factor, basic fibroblast growth factor) in supernatants and PDCs at days 3, 7, 14, and 21.

**Results:**

The SEM analysis showed a dense three-dimensional fibrin network in CGF, and CGF membranes were covered by PDCs with elongated or polygonal morphological features. Compared with the control group, CGF significantly promoted the proliferation of PDCs during the experimental period (*p* < 0.05). Immunohistochemistry revealed that PDCs were dispersedly distributed among the CGF substrates, and CD34-positive cells were also present. Moreover, CGF significantly increased the ALP activity and upregulated the expression and secretion of osteogenic differentiation and angiogenesis markers in PDCs at days 3, 7, 14, and 21 (*p* < 0.05).

**Conclusion:**

CGF can increase the proliferation and promote the osteogenic differentiation and angiogenic potential of PDCs in vitro. These results indicate that CGF can be used as a new therapeutic means for biotechnological and clinical applications.

## Introduction

Bone regeneration and reconstruction play an important role in the recovery of bone defects that do not heal spontaneously [1]. Many therapies, such as autografting and artificial bone substitutes, have been used for the improvement and treatment of bone defects during the past years [2]. However, complications, such as pain, morbidity, and functional disturbance, have hindered their clinical applications [3]. Therefore, biotechnological and bone tissue engineering strategies have developed as potential and effective alternatives [4].

Periosteum plays a crucial role in the fracture healing process, especially during the secondary callus formation. Periosteum-derived cells (PDCs) have a strong potential for proliferation and osteogenic differentiation, and they are essential for the healing and repair of bone fractures and defects [5]. Previous studies have shown that PDCs can repair a critical size long-bone defect in mice. In combination with porous scaffold, PDCs were able to treat a long bone defect in rabbits [6, 7]. In the past, the attention of bone tissue engineering and biotechnology research has been focused on the investigation of osteogenic differentiation properties of different cell types in vitro and in vivo. However, angiogenesis and timely formation of blood vessels have also played important roles in the survival of implanted cells and successful bone regeneration. Therefore, an appropriate cell source for biotechnological and bone tissue engineering applications should have both osteogenic potential and proangiogenic characteristics [8, 9]. An experimental study has shown that PDCs can produce vascular endothelial growth factor (VEGF) and exhibit proangiogenic properties [10]. As an important angiogenic factor, VEGF enhances endothelial cell proliferation, promotes cell survival under ischemic conditions, and accelerates angiogenesis and formation of the microvascular system [11]. Furthermore, PDCs contain a greater number of mesenchymal stem cells compared with bone marrow stromal cells (BMSC) [12]. Therefore, PDCs present a promising target in biotechnological and bone tissue engineering research and applications.

Concentrated growth factor (CGF), a new generation platelet concentrate, was first discovered by Sacco in 2006 [13]. Similar to the production of platelet-rich fibrin, the production process of CGF is completely natural without the addition of any biochemical agents. Therefore, CGF avoids immunoreactions, toxicity, cross-contamination, and ethical concerns. Moreover, CGF is composed of various growth factors and cytokines, such as transforming growth factor (TGF), insulin-like growth factor (IGF), platelet-derived growth factor (PDGF), and VEGF [14, 15]. Cell proliferation and osteogenic differentiation are regulated by a variety of growth factors, including bone morphogenetic protein (BMP), TGF, PDGF, IGF, core-binding factor α I, and epidermal growth factor [16]. These factors interact with each other in the process of proliferation and osteogenic differentiation, and a single factor is not sufficient to induce proliferation and osteogenesis. Many clinical studies have shown that CGF can be used as a bone graft material for hydrodynamic piezoelectric internal sinus elevation [17, 18]. Studies have also shown that CGF can increase bone formation [19] and cell differentiation [13, 20–22], as well as promoting Schwann cell proliferation and neurotrophic factor secretion [14]. However, the effects of CGF on the angiogenic potential of PDCs are still unclear.

CGF and PDCs exhibit superior clinical and biotechnological application potential. Nevertheless, studies regarding the effects of CGF on the proliferation, osteogenic differentiation, and angiogenic potential of PDCs are scarce, and their underlying mechanisms are poorly understood. In this study, we investigated the effects of CGF on the proliferation of rabbit PDCs and the expression of osteogenic differentiation markers (alkaline phosphatase (ALP), BMP-2, type I collagen (Col-1), osteocalcin (OCN), and angiogenesis markers (VEGF), basic fibroblast growth factor (bFGF)) in these cells in vitro.

## Materials and methods

### Preparation of concentrated growth factor

CGF was prepared as previously described [23]. Two milliliters of blood were drawn from the jugular vein of rabbits using Vacuette tubes (Greiner BioOne GmbH, Austria) under sterile conditions. The tubes were immediately placed in a Medifuge MF200 centrifuge (Silfradentsrl, Italy), 30 s acceleration, 500 g for 2 min, 400 g for 4 min, 500 g for 4 min, 600 g for 3 min, and 36 s deceleration. The CGF gel was collected from the middle layer of the samples (between the upper, platelet-poor plasma layer, and the lower, red blood cell layer) and pressed onto a membranous film as previously described [13]. CGF conditioned media were prepared as follows [24]: CGF membranes (each membrane was prepared from 2 mL blood) were placed in lyophilizer overnight. The lyophilized CGF membranes were ground into powder and immersed into 2 mL DMEM at 37 °C for 24 h. The media were collected and centrifuged. The supernatants were filtered by a strainer. The extracted solutions were added to 2 mL culture media and were used in the CGF group.

### Isolation and cultivation of PDCs in vitro

Thirty-six New Zealand white rabbits (male, 6–8 weeks old, 1.0–1.2 kg of body weight) were provided by the Experimental Animal Center of Jinzhou Medical University. The study protocol was reviewed and approved by the Experimental Animal Ethics Committee of Jinzhou Medical University. PDCs were isolated and cultured according to the methods described previously [10]. Femurs and tibias were isolated from each anesthetized rabbit under sterile conditions. Epiphyses were protected from digestion by submerging them in 5% low melting point agarose (SeaPlaque, Belgium). PDCs were isolated by 3 mg/mL collagenase (Sigma-Aldrich, USA) and 4 mg/mL dispase (Sigma-Aldrich, USA) in α-minimal essential medium (α-MEM) supplemented with 2 mM gluta MAX-I (Gibco, Invitrogen, USA). The first digested (10-min digestion) cell suspension was discarded as they contained cells from remaining muscles and connective tissues. PDCs were collected after a subsequent 1-h digestion. The obtained cell suspension was strained through a 70-μm cell strainer, washed, and the cell density was adjusted to 1 × 10^5^ using α-MEM containing 15% fetal bovine serum (FBS). The cells were incubated in a 5% CO_2_ atmosphere at 37 °C.

The culture medium [25] was composed of α-MEM containing 10% FBS, 100 U/mL penicillin/streptomycin, 2 mM l-glutamine, 100 μM l-ascorbate-2-phosphate, 1.8 mM inorganic phosphate, and 10^−7^ M dexamethasone (all from Gibco, Invitrogen, USA). PDCs from passages 3–4 at an initial seeding density of 2 × 10^4^ cells/cm^2^ were used for the experiments.

### Groups and observation time points

CGF conditioned media were prepared for PDCs culture. Cells were divided into two groups for Cell Counting Kit-8 (CCK-8), ALP, Western bolt, and qPCR assays: control group (PDCs + media) and CGF group (PDCs + CGF conditioned media, in which the blood and PDCs were from the same rabbit). CCK-8 assays were performed at 1, 3, 5, 7, 9, 14, and 21 days. ALP, Western bolt, and qPCR assays were performed at 3, 7, 14, and 21 days.

CGF membranes were used for the scanning electron microscopy (SEM), enzyme-linked immunosorbent assay (ELISA), and immunohistochemical staining. For SEM and immunohistochemical staining, PDCs were seeded onto CGF membranes (PDCs-CGF complex) and examined at day 4. For ELISA assay, cells were divided into two groups: control group (PDCs + media) and CGF group (PDCs + CGF membrane + media, in which the blood and PDCs were from the same rabbit), and the ELISA assays were performed at days 3, 7, 14, and 21.

### Scanning electron microscopy

The structural characterization of CGF and PDCs-CGF complex was carried out using SEM. CGF and PDCs-CGF complex were washed with phosphate-buffered saline (PBS), fixed with a 2.5% glutaraldehyde and 1% osmium tetroxide solution for 1 h, and dehydrated in a graded ethanol series followed by isoamyl acetate replacement. Afterwards, the specimens were vacuum freeze-dried, sprayed with Au, observed by S4800 SEM (Hitachi, Japan), and photographed. All experiments were performed independently and repeated three times.

### Cell proliferation assay

Proliferation of PDCs was analyzed using Cell Counting Kit-8 (CCK-8) assay (Solarbio Science & Technology, China). Three wells in each group were selected, and 10 μL of CCK-8 solution was added to each well and incubated for 2 h. A micro-plate reader (Bio-Rad, USA) was used to detect the optical density (OD) value at 450 nm. All experiments were performed independently and repeated three times.

### Alkaline phosphatase activity assay

PDCs in the control and CGF group were scraped in 0.1% Triton X-100 and sonicated. The cell lysates were centrifuged at 12,000 g for 10 min at 4 °C, and the supernatant was collected. ALP activity was determined by an ALP detection kit (Nanjing Jiancheng, China) and measuring the OD values at 520 nm according to the manufacturer’s instructions. To normalize the ALP activity, the protein concentrations were determined with the BCA assay. All experiments were performed independently and repeated three times.

### Immunohistochemical staining

PDCs-CGF complexes were washed with PBS, fixed with 4% paraformaldehyde for 1 h, embedded in paraffin blocks, and sectioned. Deparaffinized sections were incubated with 3% hydrogen peroxide for 10 min followed by antigen retrieval in 10 mM citrate buffer in a microwave oven for 10 min. The sections were treated with 0.3% Triton X-100 in PBS containing 5% bovine serum albumin to prevent nonspecific staining, blocked with 10% goat serum for 30 min, and incubated with primary anti-CD34 antibody (Biosynthesis Biotechnology, China; 1:500) at 4 °C overnight. Sections were incubated in 3,3′-diaminobenzidine for 10 min until brown color appeared. Nuclei were stained with hematoxylin for 3~5 min. All experiments were performed independently and repeated three times.

### Western blot analysis

BMP-2, Col-1, OCN, VEGF, and bFGF protein levels in the PDCs lysates were determined by Western blot analysis. The cells were homogenized using SDS lysis buffer for 30 min on ice. The protein concentration was measured by BCA assay. Then, 50 μg of total protein per sample were run on SDS-PAGE gels and transferred to PVDF membranes. The membranes were blocked with Tris-buffered saline containing 5% non-fat milk at room temperature for 2 h and incubated with the following primary antibodies at 4 °C overnight: anti-BMP-2, anti-Col-1, anti-OCN, anti-VEGF (all from Abcam, USA; 1:1000), and anti-bFGF (Biosynthesis Biotechnology, China; 1:350). After washing thrice in TBST buffer, the membranes were incubated with HRP-conjugated secondary antibody (Boster Biotechnology, China; 1:1500) for 2 h at room temperature. The chemical luminescence reaction was measured by ECL. To confirm equal protein loading, the same blots were incubated with antibodies specific for GAPDH/β-actin (Santa Cruz, USA; 1:1000). The target bands on the membranes were analyzed by a gel imaging system with the reference OD values. The experiments were performed independently and repeated three times and the relative OD values were calculated.

### Quantitative real-time reverse transcription PCR

The relative messenger ribonucleic acid (mRNA) expression of BMP-2, Col-1, OCN, VEGF, and bFGF were determined using quantitative real-time reverse transcription PCR (qRT-PCR). Total RNA was extracted from each PDCs sample using Trizol (Sigma, USA). The concentration and purity of RNA were evaluated by calculating the absorbance at 260 nm and 260/280 ratio, respectively, using an ultra-micro spectrophotometer. RT-PCR kit (TaKaRa Biotechnology, China) was used for the reverse transcription according to the manufacturer’s instructions. The primers for the target genes were listed as follows (Sangon Biotech, China): BMP-2 (forward 5′-ATAGCAGTTTCCATCACCGAA-3′, reverse 5′-ACTTCCACCACGAATCCAT-3′), Col-1 (forward 5′-GACATGTTCAGCTTTGTGGACCTC-3′, reverse 5′-GGGACCCTTAGGCCATTGTGTA-3′), OCN (forward 5′-TGAGGACCATCTTTCTGC-3′, reverse 5′- GAGCTGCTGTGACATCCATAC-3′), VEGF (forward 5′-CGAGACCTTGGTGGACATC-3′, reverse 5′-CTGCATGGTGACGTTGAAC-3′), bFGF (forward 5′-CAAGCGGCTGTACTGCAAAAA-3′, reverse 5′-AGCAAGGTAACGGTTTGCAC-3′), and GAPDH (forward 5′-ACTTTGTGAAGCTCATTTCCTGGTA-3′, reverse 5′-GTGGTTTGAGGGCTCTTACTCCTT-3′). qRT-PCR reactions were set up in 96-well plates as follows: 0.8 μL of each sense and antisense primer for the target genes, 10 μL of SYBR Premix Ex Taq II (Takara Biotechnology, China), 2 μL of cDNA, and 6.4 μL of DEPC-treated water. qRT-PCR was performed in triplicates on the Light Cycler 480 real-time PCR system (Roche Diagnostics, Germany) using the following program: initial denaturation at 95 °C for 30 s, 40 cycles of amplification at 95 °C for 5 s and 60 °C for 20 s, dissociation at 95 °C for 10 s and 65 °C for 10 s, and cooling at 40 °C for 30 s. The reference gene GAPDH was used to normalize quantitation data and show the relative expression. The expression level of the target genes was analyzed and quantified by the 2^-ΔΔ^Ct method. All experiments were performed independently and repeated three times.

### Enzyme-linked immunosorbent assay

The levels of BMP-2, Col-1, OCN, VEGF, and bFGF in supernatants were evaluated by enzyme-linked immunosorbent assay (ELISA). Briefly, PDCs cultured with or without CGF membranes were seeded onto 6-well plates. The culture supernatants were collected, and equal volumes of culture media were added to the plates. The samples were centrifuged and stored at − 80 °C until use. The ELISA assays of BMP-2, OCN, VEGF (R&D Systems, USA), Col-1, and bFGF (MultiSciences, China) were performed according to the manufacturer’s instructions. The levels of BMP-2, Col-1, OCN, VEGF, and bFGF in media and in CGF membrane + media were measured, respectively. All experiments were performed independently and repeated three times.

### Statistical analysis

All statistical analyses were performed using the SPSS 16.0 statistical package (SPSS Inc., Chicago, IL, USA) for Windows. The results were expressed as the mean ± SD. Both control and CGF intra- and inter-groups were statistically compared. The data were analyzed using Student’s *t* test and one-way ANOVA followed by post-hoc Scheffe test and multiple comparisons Dunnet *t* test. A level of *p* < 0.05 was considered to be statistically significant.

## Results

### Cellular morphology of primary PDCs

**Fig. 1:**
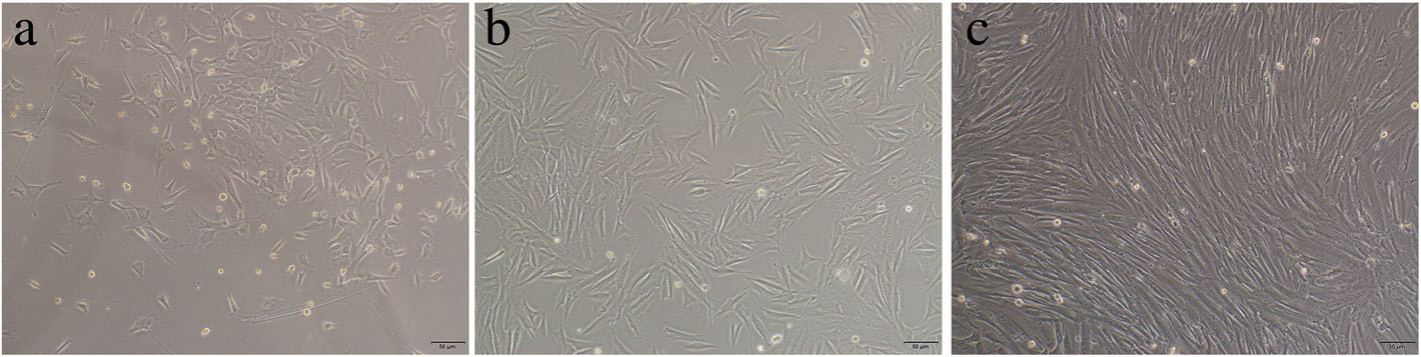
Cellular morphology of primary PDCs (magnification, × 100). **a** PDCs were irregular, spindle-shaped, and triangular at the early stage of cultivation. **b** PDCs acquired a wide, fusiform shape after 3 days of cultivation. **c** PDCs exhibited a long, spindle form and showed a whirlpool-shaped growth pattern after 7 days of cultivation. Scale bar = 50 μm. *PDCs* periosteum-derived cells. All experiments were performed independently (each experiment used PDCs and CGF from a different rabbit) and repeated three times

At the early stage of cultivation, PDCs were irregular, spindle-shaped, and triangular under inverted microscope (Fig. 1a). After 3 days of cultivation, the cells acquired a wide, fusiform shape (Fig. 1b). After 7 days of cultivation, the number of cells significantly increased, and they exhibited a long, spindle form and showed a whirlpool-shaped growth pattern (Fig. 1c).

### Morphological characterization of CGF and PDCs-CGF complex

**Fig. 2:**
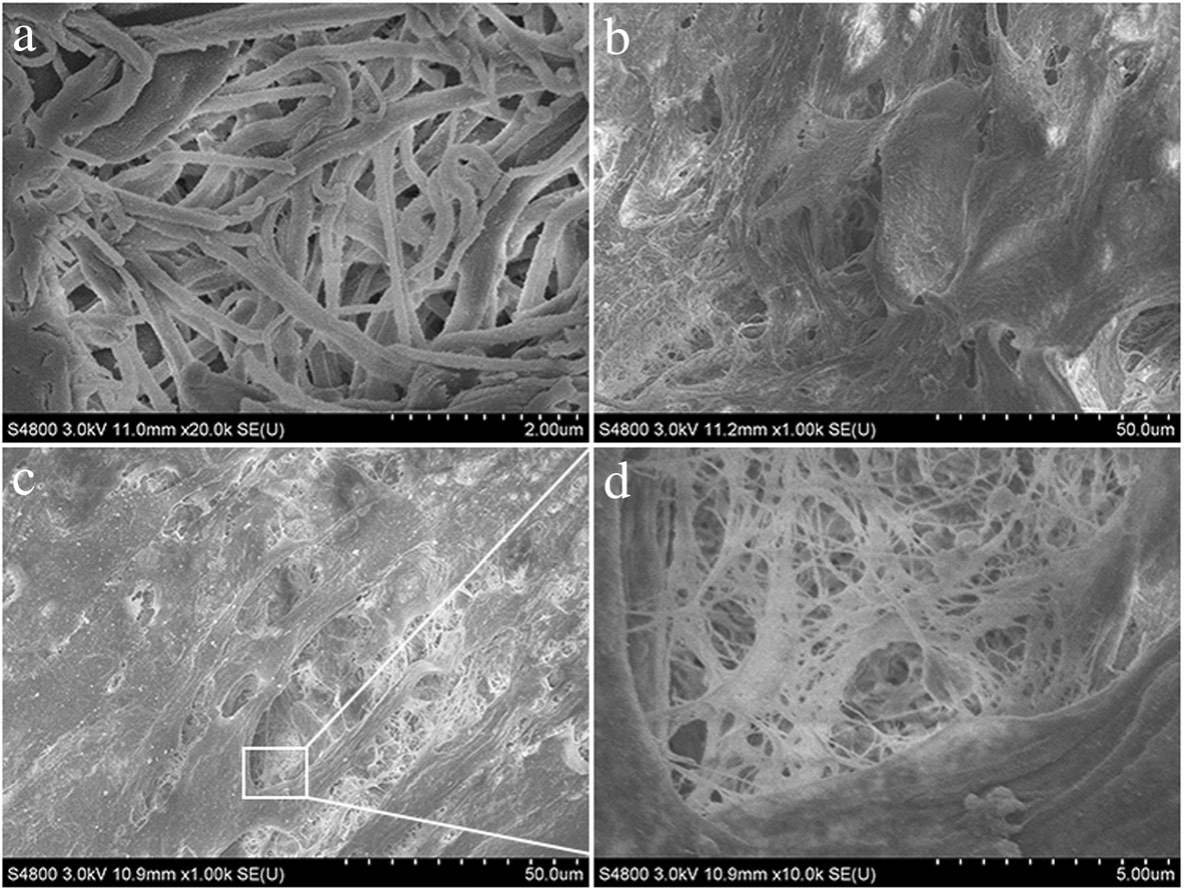
Structural characterization of CGF and PDCs-CGF complex under SEM. **a** Thin and thick fibrillar elements composed dense fibrin network in CGF. **b**, **c** CGF membrane was almost completely covered by PDCs. **d** Multiple platelet cell elements were observed in CGF. *CGF* concentrated growth factor, *PDCs* periosteum-derived cells, *SEM* scanning electron microscopy. All experiments were performed independently (each experiment used PDCs and CGF from a different rabbit) and repeated three times

A dense and porous three-dimensional fibrin network structure composed of thin and thick fibrillar elements was observed in CGF membranes using SEM analysis (Fig. 2a). After PDCs were seeded onto CGF membranes for 4 days, CGF membranes were completely covered by PDCs with elongated or polygonal morphological features (Fig. 2b, c). Multiple platelet cell elements were also observed (Fig. 2d).

### CGF significantly increased cell proliferation

**Fig. 3:**
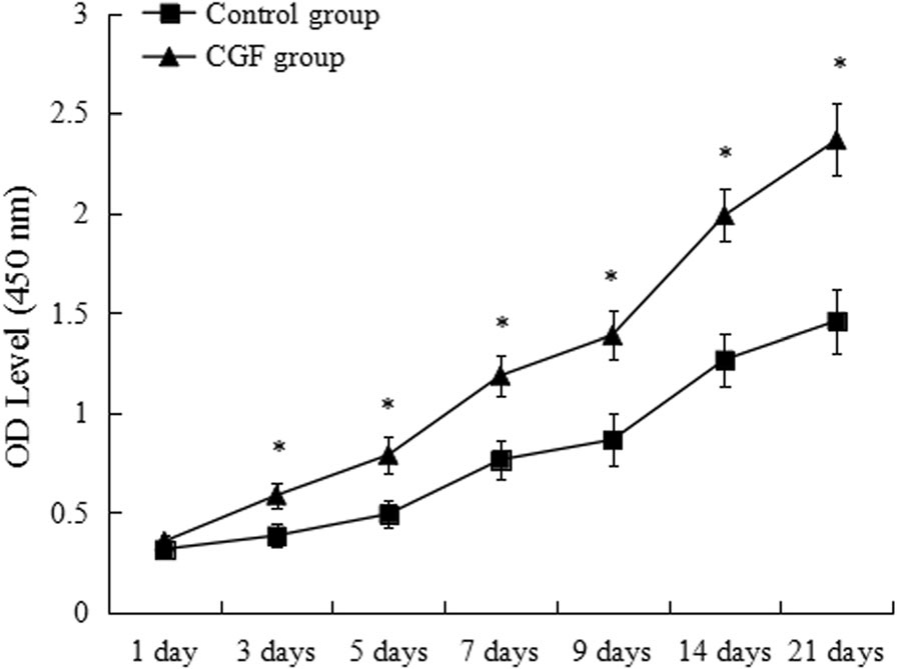
Proliferation of PDCs in control and CGF group. *p < 0.05 vs. control group. *PDCs* periosteum-derived cells, *CGF* concentrated growth factor. All data were presented as the means ± SD. All experiments were performed independently (each experiment used PDCs and CGF from a different rabbit) and repeated three times

Cell proliferation ability was evaluated by CCK-8 assay at 1, 3, 5, 7, 9, 14, and 21 days. The proliferation of PDCs increased from day 3 to day 21 in the control and CGF groups; however, the proliferation rates of PDCs were significantly higher in the CGF group compared to the control group at days 3–21(Fig. 3).

### CGF significantly increased ALP activity

**Fig. 4:**
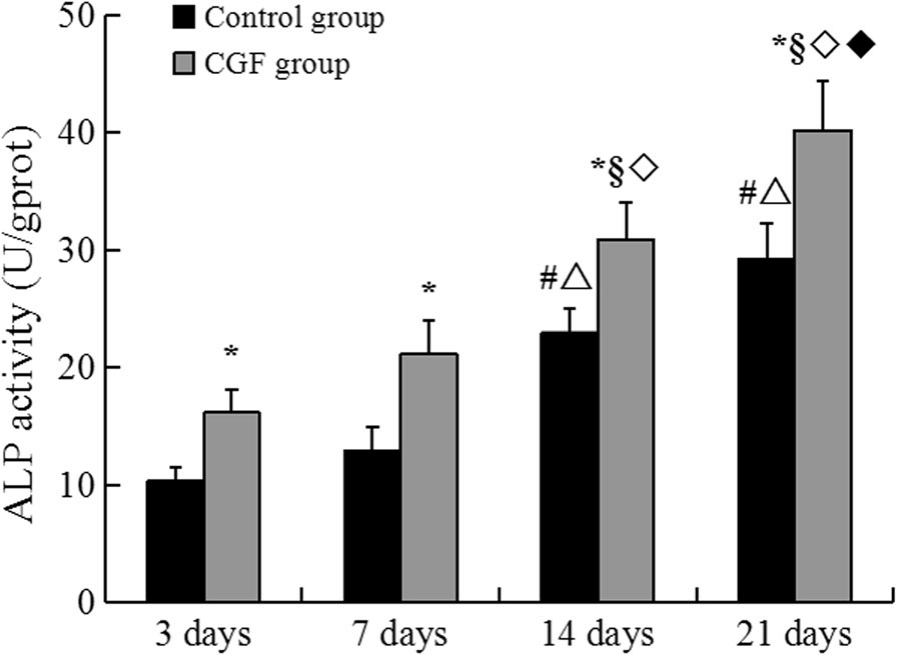
ALP activity of PDCs in control and CGF group. *p < 0.05 vs. control group. In the control intra-group: #*p* < 0.05 vs. 3 days; △*p* < 0.05 vs. 7 days. In the CGF intra-group: §*p* < 0.05 vs. 3 days; ◇*p* < 0.05 vs. 7 days; ◆*p* < 0.05 vs. 14 days. *ALP* alkaline phosphatase, *PDCs* periosteum-derived cells, *CGF* concentrated growth factor. All experiments were performed independently (each experiment used PDCs and CGF from a different rabbit) and repeated three times

In the CGF group, the ALP level on day 21 was statistically higher than days 3, 7, and 14. The ALP activity at day 14 was significantly higher compared to days 3 and 7. A similar pattern was observed in the control group, in which the ALP activity significantly increased from day 3 to day 21. The ALP levels on days 14 and 21 were statistically higher compared to the days 3 and 7, respectively. Compared with the control group, the ALP activity in the CGF group was significantly higher on days 3, 7, 14, and 21 (Fig. 4).

### Immunohistochemical staining of CD34

**Fig. 5:**
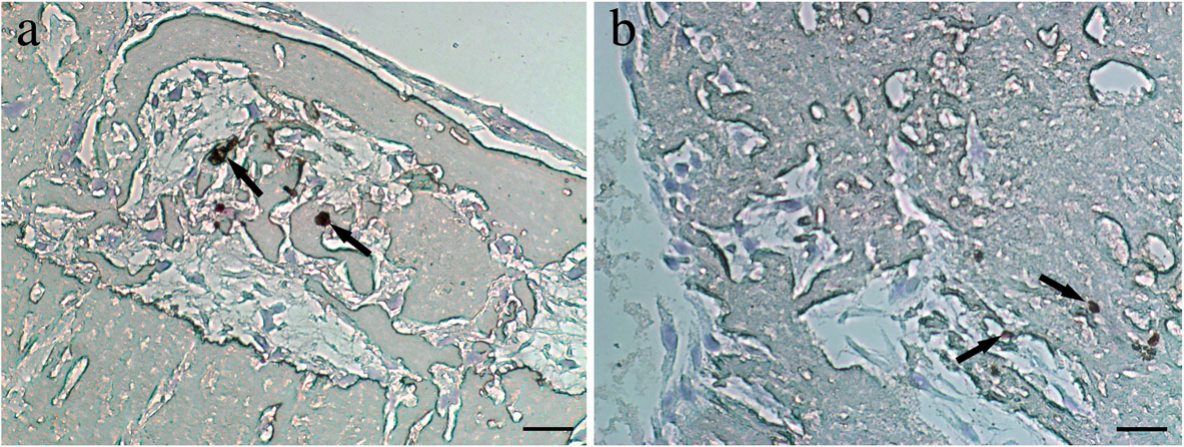
Immunohistochemical staining of CD34 in PDCs-CGF complex (magnification, × 400). PDCs were dispersedly distributed among the CGF fibrin matrix. CD34-positive cells (arrow) are visible (**a**, **b**). Scale bar = 100 μm. *CD* cluster of differentiation, *PDCs* periosteum-derived cells, *CGF* concentrated growth factor. All experiments were performed independently (each experiment used PDCs and CGF from a different rabbit) and repeated three times

The expression of CD34 in the PDCs-CGF complex was detected by immunohistochemical staining. CD34-positive cells showed brown and granular staining pattern while the nuclei of PDCs were stained blue. The results showed that PDCs were dispersedly distributed among the CGF substrates, and the presence of CD34-positive cells was observed. PDCs are CD34-negative. CGF contains CD-34 positive cells (Fig. 5).

### CGF significantly increased protein levels of BMP-2, Col-1, OCN, VEGF, and bFGF in PDCs

**Fig. 6:**
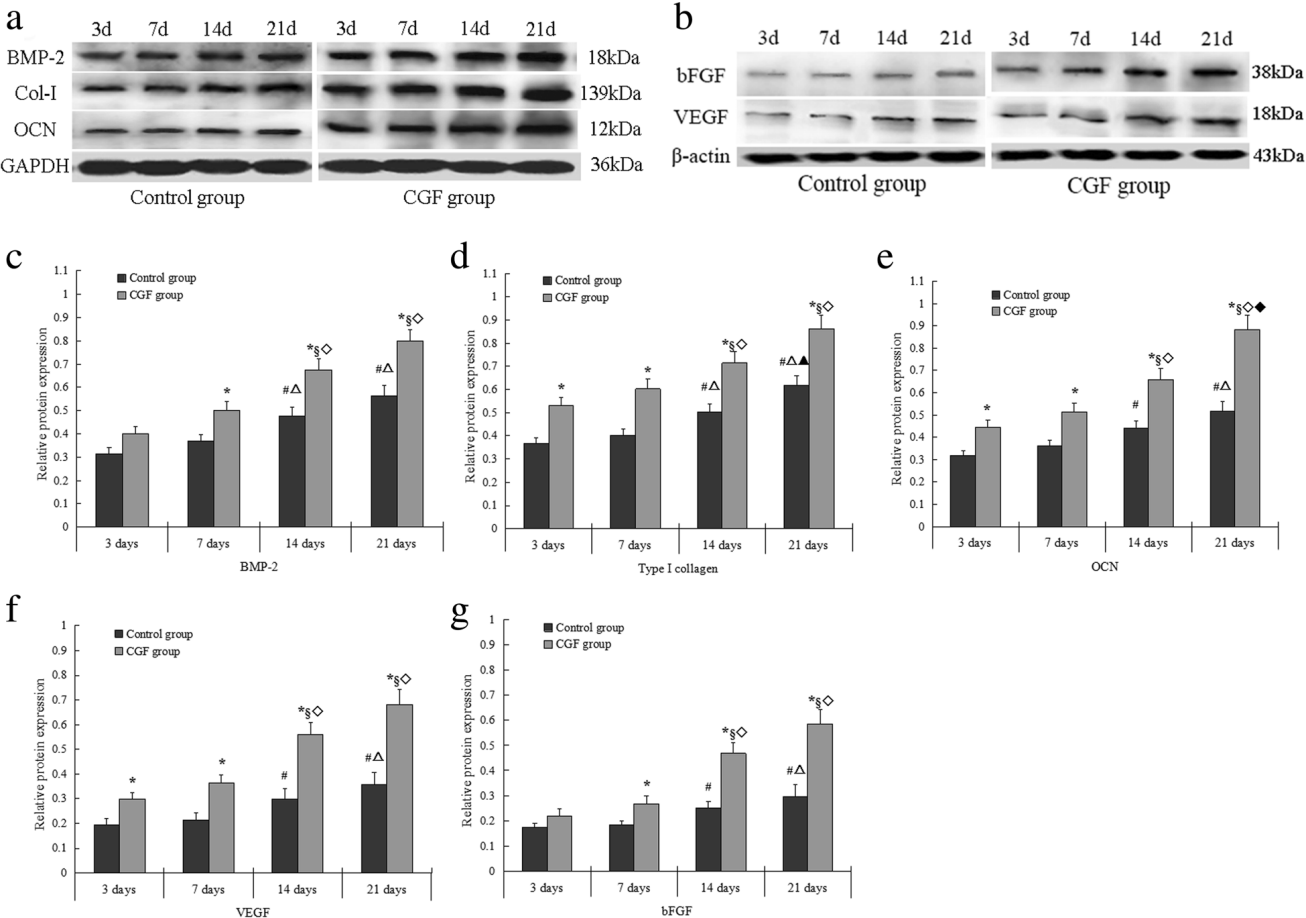
Western blot analysis for BMP-2, Col-1, OCN, VEGF, and bFGF in PDCs. **a**, **b** Representative Western blot images. **c**–**g** The quantification of protein levels of BMP-2 (**c**), Col-1 (**d**), OCN (**e**), VEGF (**f**), and bFGF (**g**) *p < 0.05 vs. control group. In control intra-group: #*p* < 0.05 vs. 3 days; △*p* < 0.05 vs. 7 days; ▲*p* < 0.05 vs. 14 days. In CGF intra-group: §*p* < 0.05 vs. 3 days; ◇*p* < 0.05 vs. 7 days; ◆*p* < 0.05 vs. 14 days. *BMP* bone morphogenetic protein, *Col*-*1* type I collagen, *OCN* osteocalcin, *VEGF* vascular endothelial growth factor, *bFGF* basic fibroblast growth factor, *PDCs* periosteum-derived cells. All data were presented as the means ± SD. All experiments were performed independently (each experiment used PDCs and CGF from a different rabbit) and repeated three times

The Western blot results showed that relative protein levels of BMP-2, Col-1, and OCN in CGF group were significantly higher than that in control group at days 3, 7, 14, and 21. Compared with the control group, the protein levels of VFGF and bFGF also significantly increased at days 3, 7, 14, and 21 in CGF group. There were differences in the protein levels of BMP-2, OCN, Col-1, VEGF, and bFGF at some timepoints in both control and CGF intra-groups (Fig. 6a–g).

### CGF significantly increased mRNA expression of BMP-2, Col-1, OCN, VEGF, and bFGF in PDCs

**Fig. 7:**
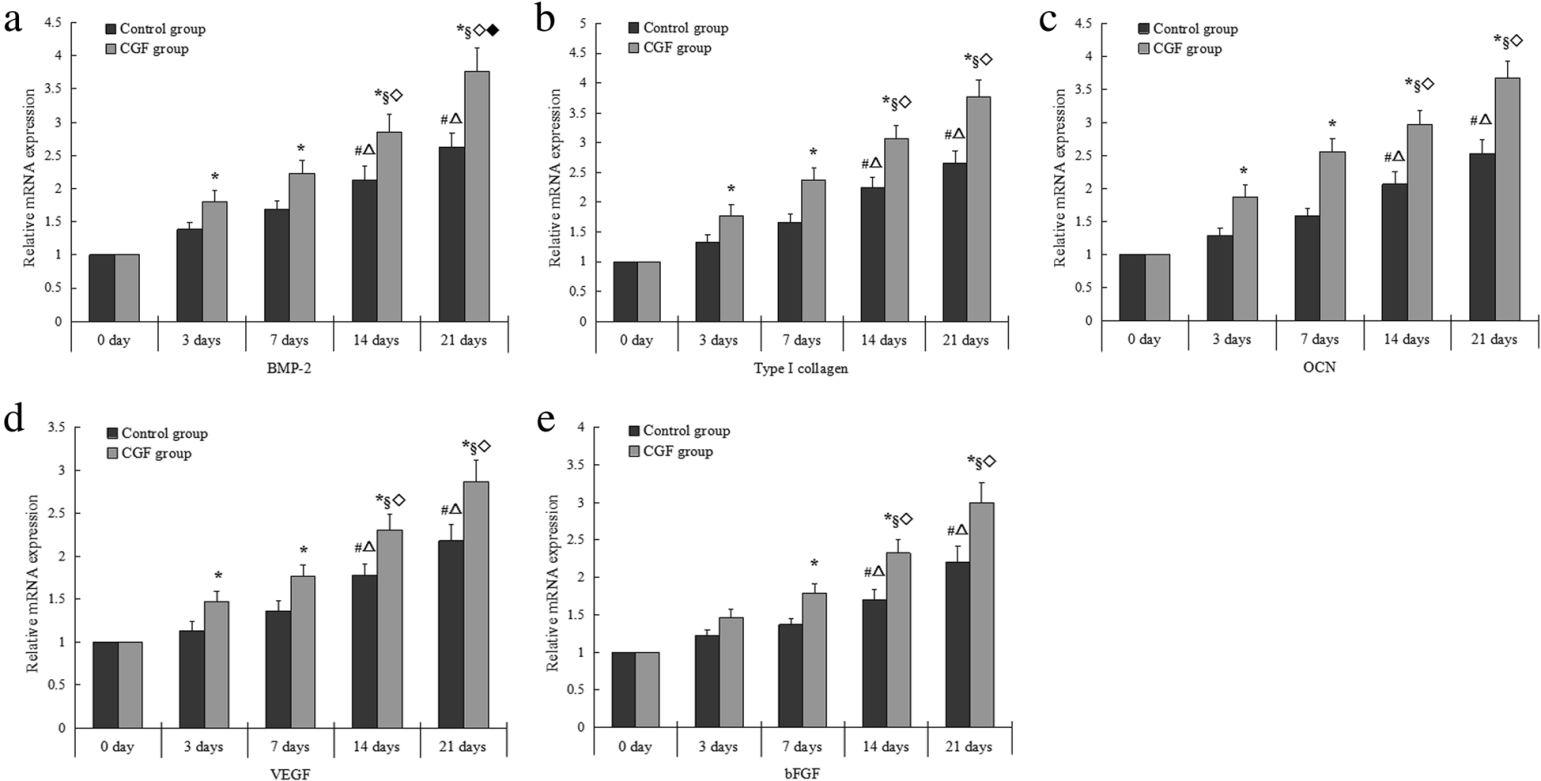
The relative mRNA expression of BMP-2 (**a**), Col-1 (**b**), OCN (**c**), VEGF (**d**), and bFGF (**e**) in PDCs. *p < 0.05 vs. control group. In control intra-group: #*p* < 0.05 vs. 3 days; △*p* < 0.05 vs. 7 days. In CGF intra-group: §*p* < 0.05 vs. 3 days; ◇*p* < 0.05 vs. 7 days; ◆*p* < 0.05 vs. 14 days. *mRNA* messenger ribonucleic acid, *BMP* bone morphogenetic protein, *Col*-*1* type I collagen, *OCN* osteocalcin, *VEGF* vascular endothelial growth factor, *bFGF* basic fibroblast growth factor, *PDCs* periosteum-derived cells. All data were presented as the means ± SD. All experiments were performed independently (each experiment used PDCs and CGF from a different rabbit) and repeated three times

Consistent with the Western blot results, a significant increase in mRNA expression of BMP-2, Col-1, OCN, and VEGF was detected in CGF group compared to that in control group at days 3, 7, 14, and 21. The mRNA expression of bFGF in CGF group significantly increased from day 7 to day 21.There were differences in mRNA expression of BMP-2, OCN, Col-1, VEGF, and bFGF at some timepoints in both control and CGF intra-groups (Fig. 7a–e).

### CGF significantly increased the secretion of BMP-2, Col-1, OCN, VEGF, and bFGF

**Fig. 8:**
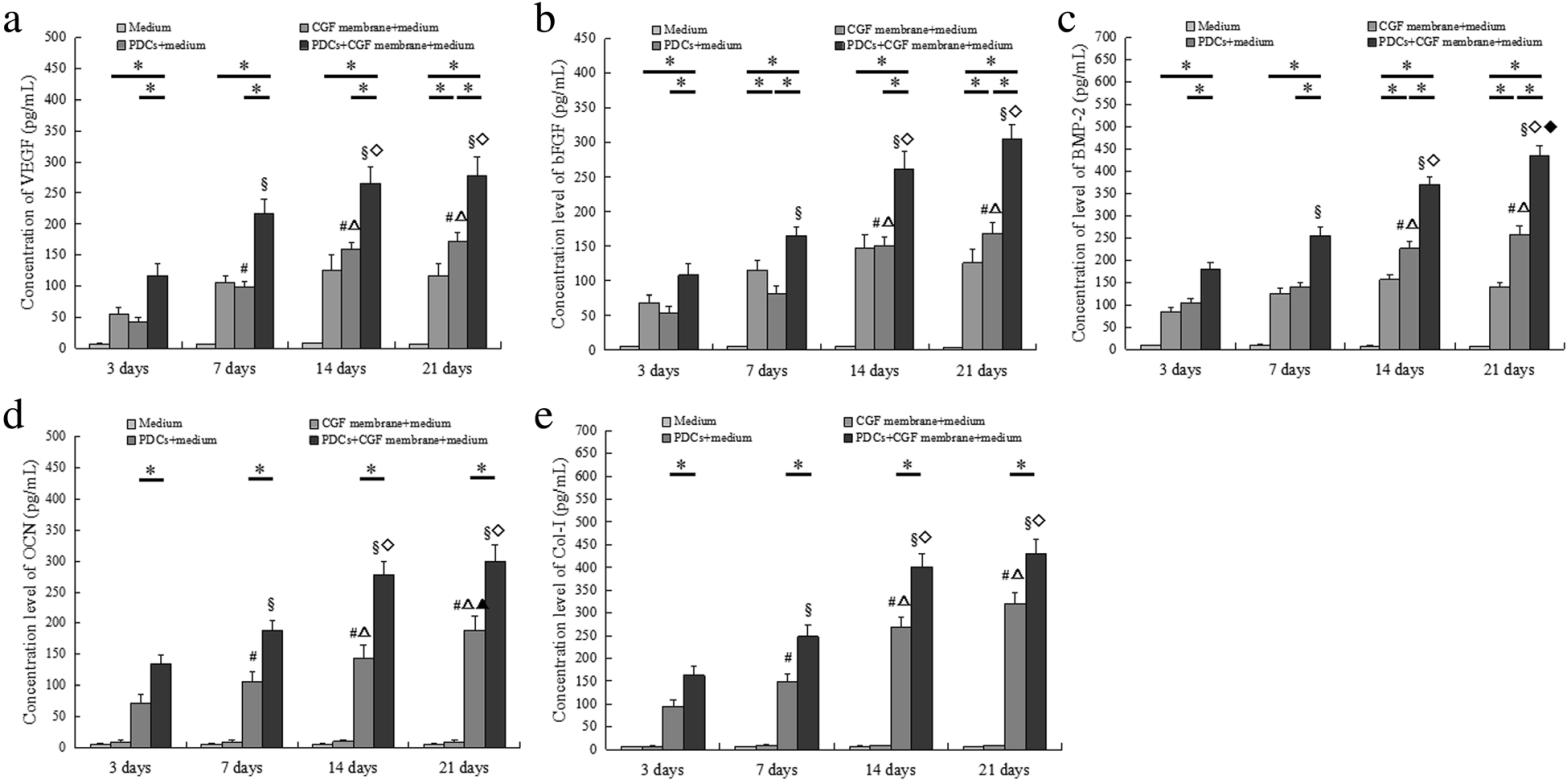
Levels of VEGF, bFGF, BMP-2, OCN, and Col-1 in supernatants were determined by ELISA. VEGF (**a**), bFGF (**b**), BMP-2 (**c**), OCN (**d**), and Col-1 (**e**) in supernatants. *p < 0.05. In the control intra-group: #*p* < 0.05 vs. 3 days; △*p* < 0.05 vs. 7 days; ▲*p* < 0.05 vs. 14 days. In the CGF intra-group: §*p* < 0.05 vs. 3 days; ◇*p* < 0.05 vs. 7 days; ◆*p* < 0.05 vs. 14 days. *ELISA* enzyme-linked immunosorbent assay, *BMP* bone morphogenetic protein, *Col*-*1* type I collagen, *OCN* osteocalcin, *VEGF* vascular endothelial growth factor, *bFGF* basic fibroblast growth factor, *PDCs* periosteum-derived cells. All data were presented as the means ± SD. All experiments were performed independently (each experiment used PDCs and CGF from a different rabbit) and repeated three times

We further detected the secretory levels of VEGF, bFGF, Col-1, OCN, and BMP-2 following CGF membrane treatment at different timepoints. We found that there was a slow release of VEGF, bFGF, and BMP2. The levels of Col-1 and OCN could not be detected in CGF membranes during the experimental period. The secretion of BMP-2, Col-1, OCN, VEGF, and bFGF increased from day 3 to day 21 in both control and CGF groups. Compared with the control group, the secretion of BMP-2, Col-1, OCN, VEGF, and bFGF significantly increased in the CGF group at the same timepoints. There were differences in the secretory levels of these factors at some timepoints in both control and CGF intra-groups (Fig. 8a–e).

## Discussion

Periosteum contains a population of multipotent mesenchymal progenitor cells (bone and cartilage precursor cells), which play a crucial role during the process of fracture healing, bone defect repair, and regeneration [26]. PDCs are known to possess a higher number of MSCs than BMSCs. PDCs not only have a stronger osteogenic potential in vivo and in vitro but also display marked proangiogenic properties [27]. In this study, PDCs were isolated from the femurs and tibias of 6–8-week-old New Zealand rabbits. These PDCs exhibited a fibroblast-like morphology and had a high proliferative capacity during the experimental period.

Our SEM analysis indicates that CGF has a complex three-dimensional fibrin structure. This dense fibrin network can provide a matrix for cell adherence and migration. More importantly, the three-dimensional fibrin structure is crucial for blood vessel infiltration and nutrients diffusion. It is known that cell proliferation can increase cell population necessary for filling the wound in a bone defect site at all stages of wound healing and bone regeneration. In this study, we observed a significant increase of PDCs proliferation from day 3 to day 21 in CGF groups. CGF contains a larger fibrin matrix and higher levels of growth factors in comparison to the platelet concentrates. During the cell cultivation, VEGF, bFGF, and BMP-2were released from CGF for a relatively longer period, which could promote cell proliferation and enhance cell activity. A previous study has indicated that many platelet concentrate products have toxic and inhibitory effects on the cultured cells [28]. However, we did not find cytotoxicity and inhibitory effect of CGF on PDCs during the experimental period.

Osteogenic differentiation is a multi-step process that is controlled by complex molecular mechanisms and factors. Col-1, ALP, and OCN are produced or synthesized in different cell phase or state, and is closely associated with osteogenic differentiation and calcification during bone formation [29, 30]. All the above factors are considered to be the major markers that determine stem cell differentiation into osteoblasts. In this study, CGF increased the ALP activity of PDCs during the experimental period. Furthermore, the expression of Col-1 and OCN in PDCs significantly increased by CGF from day 3 to day 21 as determined using Western blot and qRT-PCR analyses. These results indicate that CGF can enhance the osteogenic activity of PDCs in a time-dependent manner. Similarly, a previous study has also shown that CGF induces beagle periodontal ligament stem cell differentiation into osteoblasts in vitro and accelerates osteogenesis during the differentiation process [13]. BMP belongs to the TGF-β family, and it possesses the strongest potential among BMP family members to induce bone formation [31]. In this study, the Western blot results showed that CGF upregulated BMP-2 protein level in PDCs. We further determined whether PDCs express BMP-2 gene using qRT-PCR. We found that BMP-2 mRNA expression was low in the control group; while CGF increased BMP-2 mRNA expression in PDCs from day 3 to day 21. This study not only detected the expression of Col-1, OCN, and BMP-2 on the intracellular level but also investigated the secretion of Col-1, OCN, and BMP-2 following CGF membrane treatment to investigate the underlying mechanisms of CGF-induced PDCs differentiation. We did not detect Col-1 and OCN secretion in CGF membrane during the experimental period, whereas BMP-2 was gradually released and significantly increased at days 7 and 14. The secretion of BMP2, Col-1, and OCN increased from day 3 to day 21 in the control group. CGF significantly increased BMP2, Col-1, and OCN secretion during the experimental period. PDCs can secrete BMP-2, Col-1, and OCN while other factors, such as BMP-2, released by CGF can further promote the osteogenic differentiation of PDCs. CGF is rich in a variety of growth factors and cytokines, and these factors can exhibit a synergistic effect in promoting the expression of osteogenic differentiation markers (ALP, Col-1, OCN, and BMP-2) through cell-membrane-receptor-mediated, autocrine, or paracrine signaling pathways during the process of osteogenic differentiation and bone regeneration. Our study confirmed that CGF promotes the osteogenic differentiation of PDCs in vitro.

As mentioned above, CGF can promote bone formation and cell differentiation [19–22]. However, most of these studies have focused on the cell osteogenic characteristics. There are few studies on angiogenic potential of cells. Another important aim of this study was to evaluate the effects of CGF on the angiogenic potential of PDCs. It is known that the newly generated blood vessels can provide nutrients and accelerate bone fracture healing. The major growth factors that are crucial in angiogenesis include bFGF and VEGF. bFGF is an angiogenic growth factor that stimulates endothelial cell proliferation and migration. It is also an efficient mediator of VEGF activity, which plays a key role in the regulation of angiogenesis as well as cell survival, division, differentiation, and migration [32]. Numerous studies have indicated that VEGF accumulation within a bone fracture site attracts osteoprogenitor and endothelial cells, creating an environment where these distinct cell types secrete trophic factors that mutually promote their proliferation and survival [33]. Rodella et al. found that there are CD34-positive cells in CGF and RBC layers, especially in the CGF layers [23]. The immunohistochemical staining results of our study also showed the presence of CD34-positive cells that were dispersedly distributed among the fibrin network of a CGF membrane. Circulating CD34-positive cells are involved in vascular maintenance, neovascularization, and angiogenesis. Furthermore, the expression and secretion of VEGF and bFGF in PDCs upregulated at days 3, 7, 14, and 21 after the treatment of CGF conditioned media or CGF membranes. The culture system serves as a microenvironment where CGF provides a variety of growth factors, and the fibrin network structure ensures the presence of CD34-positive cells and stimulates the expression of bFGF and VEGF in PDCs. These findings indicate that CGF can promote the angiogenic potential of PDCs in vitro. The increasing number of findings suggests that angiogenesis and osteogenesis are mutually interdependent. Additionally, many in vitro studies have shown that VEGF treatment can increase the expression of osteoblastic differentiation markers and accelerate osteoblast differentiation of periosteal cells and human periosteum-derived stem cells [34, 35]. A cross-talk between angiogenesis and osteogenesis during the process of osteoblastic differentiation has attracted a significant amount of attention and deserves further research in bone tissue engineering.

## Conclusion

In conclusion, CGF as a source of growth factors and a supportive fibrin matrix can promote the proliferation of PDCs and increase the expression of osteogenic differentiation and angiogenesis markers in these cells in vitro. Our study demonstrates the promising effects of CGF on PDCs, which provides an experimental basis for further exploration of their clinical application in bone tissue engineering. Future studies are necessary to investigate these effects in vivo and examine the underlying biological mechanisms in more details.

## Abbreviations

ALP: Alkaline phosphatase
bFGF: Basic fibroblast growth factor
BMP: Bone morphogenetic protein
BMSC: Bone marrow stromal cells
CCK-8: Cell Counting Kit-8
CD: Cluster of differentiation
CGF: Concentrated growth factor
Col-1: Type I collagen
ELISA: Enzyme-linked immunosorbent assay
FBS: Fetal bovine serum
IGF: Insulin-like growth factor
mRNA: Messenger ribonucleic acid
OCN: Osteocalcin
OD: Optical density
PBS: Phosphate-buffered saline
PDCs: Periosteum-derived cells
PDGF: Platelet-derived growth factor
qRT-PCR: Quantitative real-time reverse transcription PCR
SEM: Scanning electron microscopy
TGF: Transforming growth factor
VEGF: Vascular endothelial growth factor
α-MEM: α-Minimal essential medium

## References

[1] Agata H, Asahina I, Yamazaki Y, Uchida M, Shinohara Y, Honda MJ, Kagami H, Ueda M (2007). Effective bone engineering with periosteum-derived cells. J Dent Res.

[2] De Santis E, Lang NP, Cesaretti G, Mainetti T, Beolchini M, Botticelli D (2013). Healing outcomes at implants installed in sites augmented with particulate autologous bone and xenografts. An experimental study in dogs. Clin Oral Implants Res.

[3] Cicciu M, Cervino G, Herford AS, Fama F, Bramanti E, Fiorillo L, Lauritano F, Sambataro S, Troiano G, Laino L. Facial bone reconstruction using both marine or non-marine bone substitutes: evaluation of current outcomes in a systematic literature review. Marine drugs. 2018;16(1). 10.3390/md16010027 PMC5793075 29342834

[4] Claudia DF, Manuela M, Alberto RC, Marila C, Luigia S, Baena RRY, Cesare P (2015). Stem cells and translational medicine: from research to clinical procedures. Current Tissue Engineering.

[5] Filion TM, Song J (2013). A sulfated nanofibrous mesh supporting the osteogenic differentiation of periosteum-derived cells. Journal of biomaterials and tissue engineering.

[6] Bolander J, Ji W, Leijten J, Teixeira LM, Bloemen V, Lambrechts D, Chaklader M, Luyten FP (2017). Healing of a large long-bone defect through serum-free in vitro priming of human periosteum-derived cells. Stem cell reports.

[7] Bakker AD, Schrooten J, van Cleynenbreugel T, Vanlauwe J, Luyten J, Schepers E, Dubruel P, Schacht E, Lammens J, Luyten FP (2008). Quantitative screening of engineered implants in a long bone defect model in rabbits. Tissue engineering Part C, Methods.

[8] Au P, Tam J, Fukumura D, Jain RK (2008). Bone marrow-derived mesenchymal stem cells facilitate engineering of long-lasting functional vasculature. Blood.

[9] Unger RE, Sartoris A, Peters K, Motta A, Migliaresi C, Kunkel M, Bulnheim U, Rychly J, Kirkpatrick CJ (2007). Tissue-like self-assembly in cocultures of endothelial cells and osteoblasts and the formation of microcapillary-like structures on three-dimensional porous biomaterials. Biomaterials.

[10] van Gastel N, Torrekens S, Roberts SJ, Moermans K, Schrooten J, Carmeliet P, Luttun A, Luyten FP, Carmeliet G (2012). Engineering vascularized bone: osteogenic and proangiogenic potential of murine periosteal cells. Stem Cells.

[11] Street J, Bao M, deGuzman L, Bunting S, Peale FV, Ferrara N, Steinmetz H, Hoeffel J, Cleland JL, Daugherty A (2002). Vascular endothelial growth factor stimulates bone repair by promoting angiogenesis and bone turnover. Proc Natl Acad Sci U S A.

[12] Yoshimura H, Muneta T, Nimura A, Yokoyama A, Koga H, Sekiya I (2007). Comparison of rat mesenchymal stem cells derived from bone marrow, synovium, periosteum, adipose tissue, and muscle. Cell Tissue Res.

[13] Yu B, Wang Z (2014). Effect of concentrated growth factors on beagle periodontal ligament stem cells in vitro. Mol Med Rep.

[14] Qin J, Wang L, Sun Y, Sun X, Wen C, Shahmoradi M, Zhou Y (2016). Concentrated growth factor increases Schwann cell proliferation and neurotrophic factor secretion and promotes functional nerve recovery in vivo. Int J Mol Med.

[15] Qiao J, An N, Ouyang X (2017). Quantification of growth factors in different platelet concentrates. Platelets.

[16] Ducy P, Zhang R, Geoffroy V, Ridall AL, Karsenty G (1997). Osf2/Cbfa1: a transcriptional activator of osteoblast differentiation. Cell.

[17] Kim JM, Sohn DS, Bae MS, Moon JW, Lee JH, Park IS (2014). Flapless transcrestal sinus augmentation using hydrodynamic piezoelectric internal sinus elevation with autologous concentrated growth factors alone. Implant Dent.

[18] Sohn DS, Heo JU, Kwak DH, Kim DE, Kim JM, Moon JW, Lee JH, Park IS (2011). Bone regeneration in the maxillary sinus using an autologous fibrin-rich block with concentrated growth factors alone. Implant Dent.

[19] Kim TH, Kim SH, Sandor GK, Kim YD (2014). Comparison of platelet-rich plasma (PRP), platelet-rich fibrin (PRF), and concentrated growth factor (CGF) in rabbit-skull defect healing. Arch Oral Biol.

[20] Bonazza V, Borsani E, Buffoli B, Parolini S, Inchingolo F, Rezzani R, Rodella LF (2018). In vitro treatment with concentrated growth factors (CGF) and sodium orthosilicate positively affects cell renewal in three different human cell lines. Cell Biol Int.

[21] Hong S, Chen W, Jiang B (2018). A comparative evaluation of concentrated growth factor and platelet-rich fibrin on the proliferation, migration, and differentiation of human stem cells of the apical papilla. J Endod.

[22] Chen X, Wang J, Yu L, Zhou J, Zheng D, Zhang B (2018). Effect of concentrated growth factor (CGF) on the promotion of osteogenesis in bone marrow stromal cells (BMSC) in vivo. Sci Rep.

[23] Rodella LF, Favero G, Boninsegna R, Buffoli B, Labanca M, Scari G, Sacco L, Batani T, Rezzani R (2011). Growth factors, CD34 positive cells, and fibrin network analysis in concentrated growth factors fraction. Microsc Res Tech.

[24] Ling Z, Lin W, Jie Q, Sun X, Yang T, Ni Y, Zhou Y (2015). New biodegradable implant material containing hydrogel with growth factors of lyophilized PRF in combination with an nHA/PLGA scaffold. Journal of Hard Tissue Biology.

[25] Mrozik K, Gronthos S, Shi S, Bartold PM (2010). A method to isolate, purify, and characterize human periodontal ligament stem cells. Methods Mol Biol.

[26] Malizos KN, Papatheodorou LK (2005). The healing potential of the periosteum molecular aspects. Injury.

[27] Mrosek EH, Schagemann JC, Chung HW, Fitzsimmons JS, Yaszemski MJ, Mardones RM, O'Driscoll SW, Reinholz GG (2010). Porous tantalum and poly-epsilon-caprolactone biocomposites for osteochondral defect repair: preliminary studies in rabbits. Journal of orthopaedic research : official publication of the Orthopaedic Research Society.

[28] Graziani F, Ivanovski S, Cei S, Ducci F, Tonetti M, Gabriele M (2006). The in vitro effect of different PRP concentrations on osteoblasts and fibroblasts. Clin Oral Implants Res.

[29] Lin HT, Tarng YW, Chen YC, Kao CL, Hsu CJ, Shyr YM, Ku HH, Chiou SH (2005). Using human plasma supplemented medium to cultivate human bone marrow-derived mesenchymal stem cell and evaluation of its multiple-lineage potential. Transplant Proc.

[30] Higuchi C, Nakamura N, Yoshikawa H, Itoh K (2009). Transient dynamic actin cytoskeletal change stimulates the osteoblastic differentiation. J Bone Miner Metab.

[31] Khan SN, Bostrom MP, Lane JM (2000). Bone growth factors. The Orthopedic clinics of North America.

[32] Han SK, Chun KW, Gye MS, Kim WK (2006). The effect of human bone marrow stromal cells and dermal fibroblasts on angiogenesis. Plast Reconstr Surg.

[33] Beamer B, Hettrich C, Lane J (2010). Vascular endothelial growth factor: an essential component of angiogenesis and fracture healing. HSS journal : the musculoskeletal journal of Hospital for Special Surgery.

[34] Ferretti C, Borsari V, Falconi M, Gigante A, Lazzarini R, Fini M, Di Primio R, Mattioli-Belmonte M (2012). Human periosteum-derived stem cells for tissue engineering applications: the role of VEGF. Stem Cell Rev.

[35] Samee M, Kasugai S, Kondo H, Ohya K, Shimokawa H, Kuroda S (2008). Bone morphogenetic protein-2 (BMP-2) and vascular endothelial growth factor (VEGF) transfection to human periosteal cells enhances osteoblast differentiation and bone formation. J Pharmacol Sci.

